# Guidance for the treatment and prevention of obstetric-associated venous thromboembolism

**DOI:** 10.1007/s11239-015-1309-0

**Published:** 2016-01-16

**Authors:** Shannon M. Bates, Saskia Middeldorp, Marc Rodger, Andra H. James, Ian Greer

**Affiliations:** Department of Medicine, McMaster University and Thrombosis and Atherosclerosis Research Institute (TaARI), 1280 Main Street West, HSC 3W11, Hamilton, ON L8S 4K1 Canada; Department of Vascular Medicine, University of Amsterdam, Amsterdam, The Netherlands; Departments of Medicine, Epidemiology and Community Medicine, and Obstetrics and Gynecology, University of Ottawa, Ottawa, ON Canada; Department of Obstetrics and Gynecology, Duke University, Durham, NC USA; Faculty of Medical and Human Sciences, The University of Manchester, Manchester, UK

**Keywords:** Pregnancy, Obstetric, Venous thromboembolism, Pulmonary embolism, Deep vein thrombosis, Prophylaxis, Anticoagulants

## Abstract

Venous thromboembolism (VTE), which may manifest as pulmonary embolism (PE) or deep vein thrombosis (DVT), is a serious and potentially fatal condition. Treatment and prevention of obstetric-related VTE is complicated by the need to consider fetal, as well as maternal, wellbeing when making management decisions. Although absolute VTE rates in this population are low, obstetric-associated VTE is an important cause of maternal morbidity and mortality. This manuscript, initiated by the Anticoagulation Forum, provides practical clinical guidance on the prevention and treatment of obstetric-associated VTE based on existing guidelines and consensus expert opinion based on available literature where guidelines are lacking.

## Introduction

Venous thromboembolism (VTE), which may manifest as pulmonary embolism (PE) or deep vein thrombosis (DVT), complicates 0.5–2.2 per 1000 deliveries, depending on the population studied [1–8]. During pregnancy, the risk of VTE is increased five to tenfold compared to non-pregnant women of comparable age [1, 9, 10]. The postpartum period poses a higher risk [1, 7, 10] and during this time frame, the daily risk of VTE is increased 15- to 35-fold compared to age-matched non-pregnant women [1, 9]. The daily risk of pregnancy-associated VTE appears greatest during the first 3–6 weeks postpartum [1, 7]. After that, the risk declines rapidly, although a small residual risk increase may persist for 12 weeks after delivery [7, 10, 11]. Although the absolute VTE rates are low, pregnancy-associated VTE is an important cause of maternal morbidity [12–14] and mortality [15, 16].

The treatment and prevention of pregnancy-associated VTE is challenging because of the potential for both fetal and maternal complications, as well as the paucity of relevant high quality research. Although evidence-based guideline recommendations for the use of anticoagulants in this patient population have been published [17–25], they are based largely upon observational studies and extrapolated from data in non-pregnant patients. The lack of high quality data specific to pregnancy results in a lack of consistency in their recommendations. This chapter reviews the published evidence base and uses that information, as well as published guidelines, to provide practical guidance for the management and prevention of VTE during pregnancy.

## Methods

The goal of this chapter is to provide guidance to providers on how best to individualize care to patients with pregnancy-associated VTE, with specific focus on the questions listed in Table 1. Questions were developed by consensus from the authors. To address these questions, current guidelines from the American College of Obstetricians and Gynecologists (ACOG) [17, 18], the Society of Obstetricians and Gynaecologists of Canada (SOGC) [19], the Royal College of Obstetricians and Gynaecologists (RCOG) [20, 21], clinicians from Australia and New Zealand [22], and American College of Chest Physicians (ACCP) [23–25] were reviewed and relevant recommendations were extracted (Tables 2A–2D). The literature was reviewed and data from relevant systematic reviews, randomized trials, and observational studies were incorporated. The authors’ consensus interpretation of these studies, in the context of the realities of VTE care, was distilled into the practical recommendations that are presented in this article.

**Table 1:** Guidance questions to be considered

1. What are the risks of anticoagulant use during pregnancy?
2. What are the risks of anticoagulation in breastfeeding women?
3. How is venous thromboembolism during pregnancy treated?
4. How is pregnancy-associated VTE prevented?
5. How is peripartum anticoagulation managed?

**Table 2A:** Guideline summary—anticoagulant choice

American College of Obstetricians and Gynecologists (ACOG) [17, 18]	Society of Obstetricians and Gynaecologists of Canada (SOGC) [19]	Royal College of Obstetricians and Gynaecologists (RCOG) [20, 21]	Australia/New Zealand [22]	American College of Chest Physicians (ACCP) [23]
During pregnancy				
Heparin compounds are the preferred anticoagulant during pregnancy **(Level B)**	LMWH is the preferred pharmacologic agent over UFH for treatment of VTE during pregnancy **(II-2A)** LMWH is the preferred pharmacologic agent over UFH for antepartum thromboprophylaxis **(III-A)** LMWH is the preferred pharmacologic agent over UFH for postpartum thromboprophylaxis **(IIIA)** Vitamin K antagonists should only be considered for treatment of VTE in exceptional circumstances **(II-2A)** Recommend against the use of oral Xa inhibitors and oral direct thrombin inhibitors **(III-D)**	LMWH is the preferred anticoagulant for treatment of acute VTE during pregnancy **(B)** LMWHs are the agents of choice for antenatal and postnatal thromboprophylaxis **(A)** Because of their adverse effects on the fetus, vitamin K antagonists should not be used for antenatal VTE treatment **(C)** Women receiving long-term vitamin K antagonist therapy should be counselled about the risks of vitamin K antagonists to the fetus and advised to stop these medications and change to LMWH as soon as pregnancy is confirmed (ideally within 2 weeks of the missed period and before the 6th week of pregnancy) **(no grade)** Oral thrombin and Xa inhibitors should be avoided in pregnant women **(no grade)**	Women with VTE in pregnancy should not be treated with vitamin K antagonists, such as warfarin **(Consensus Level 1)**	For pregnant patients, recommend LMWH for prevention and treatment of VTE, instead of UFH **(Grade 1B)** For pregnant women, recommend avoiding the use of oral direct thrombin and factor Xa inhibitors **(Grade 1C)** For women requiring long-term vitamin K antagonists who are attempting pregnancy and are candidates for LMWH substitution, suggest performing frequent pregnancy tests and substituting LMWH for vitamin K antagonists when pregnancy is achieved rather than switching to LMWH while attempting pregnancy **(Grade 2C)** [Remark: Women who place little value on avoiding the risks, inconvenience, and costs of LMWH therapy of uncertain duration while awaiting pregnancy and a high value on minimizing the risks of early miscarriage associated with vitamin K antagonist therapy are likely to choose LMWH while attempting pregnancy]
During pregnancy if HIT or other heparin allergy				
Fondaparinux is preferred if there is severe cutaneous heparin allergy or HIT **(no grade)**	Consultation with a hematologist or thrombosis specialist is recommended to consider the use of heparinoids if HIT occurs **(II-3B)**	Pregnant women who develop HIT or have heparin allergy and require continuing anticoagulant therapy should be managed an alternative anticoagulant under specialist advice **(C)** Consideration should be given to the use of fondaparinux, argatroban, or r-hirudin in pregnant women who are unable to tolerate heparin, UFH or danaparoid and who require continuing anticoagulant therapy **(D)**		For pregnant women, suggest limiting the use of fondaparinux and parenteral direct thrombin inhibitors to those with severe allergic reactions to heparin (e.g. HIT) who cannot receive danaparoid **(Grade 1C)**
During breastfeeding				
Warfarin, LMWH, unfractionated heparin are compatible with breast feeding **(Level B)**		Women should be advised that neither UFH, LMWH nor warfarin is contraindicated in breastfeeding **(D)**		For lactating women using warfarin, acenocoumarol or UFH who wish to breastfeed, recommend continuing the use of warfarin, acenocoumarol, or UFH **(Grade 1A)** For lactating women using LMWH, danaparoid, or r-hirudin who wish to breastfeed, recommend continuing the use of LMWH, danaparoid, or r-hirudin **(Grade 1B)** For breastfeeding women, suggest alternative anticoagulants rather than fondaparinux **(Grade 2C)** For breastfeeding women, we recommend alternative anticoagulants rather than oral direct thrombin and factor Xa inhibitors **(Grade 1C)** For lactating women using low-dose Aspirin for vascular indications who wish to breastfeed, we suggest continuing this medication **(Grade 1B)**

Please see individual references for grading criteria

**Table 2B:** Guideline summary—management of acute venous thromboembolism

American College of Obstetricians and Gynecologists (ACOG) [17, 18]	Society of Obstetricians and Gynaecologists of Canada (SOGC) [19]	Royal College of Obstetricians and Gynaecologists (RCOG) [20, 21]	Australia/New Zealand [22]	American College of Chest Physicians (ACCP) [23]
Hospitalization				
Hospitalization in cases of hemodynamic instability, large VTE or maternal co-morbidity **(no grade)**	Pregnant women with acute VTE should be hospitalized or followed closely as outpatients for the first 2 weeks after diagnosis **(III-C)**		Inpatient observation and treatment of pregnant women with PE for the first few days following diagnosis is recommended **(Consensus Level 1)**	
Anticoagulation				
Therapeutic anticoagulation during current pregnancy **(Level C)**	Manufacturer’s recommendations for LMWH dosing based on patient’s current weight should be adhered to **(II-1A)** LMWH can be administered once or twice a day depending on the agent selected **(III-C)** Following initial treatment, anticoagulant intensity can be decreased to intermediate or prophylactic dose for the remainder of the pregnancy and for at least 6 weeks postpartum **(III-C)**	LMWH be given daily in the women’s booking early pregnancy weight, There is insufficient evidence to recommend whether the dose of LMWH should be given once daily or in two divided doses (**C)**	Treatment of acute VTE in pregnancy should be with LMWH given once-daily or twice-daily at therapeutic doses. There is currently insufficient evidence to favour one dose regimen over the other **(Group Consensus Level 1)** Women with PE or more extensive DVT (i.e. iliofemoral thrombosis during pregnancy should receive twice-daily LMWH for at least 8–12 weeks, after which time a reduction to a once-daily regimen may be considered **(Consensus Level 2)**	For pregnant women with acute VTE, recommend therapy with weight adjusted-dose subcutaneous LMWH over adjusted-dose UFH **(Grade 1B)** A decrease in dosing intensity later in therapy to 75 % of a full-treatment dose may be useful in women at risk of anticoagulant-related bleeding or heparin-associated osteoporosis **(no grade)**
Intravenous UFH can be considered in the initial treatment of PE and in situations in which delivery, surgery, or thrombolysis may be necessary **(no grade)**		Intravenous UFH is the preferred treatment in massive PE with cardiovascular collapse **(B)** Consideration should be given to the use of intravenous UFH when VTE occurs at term **(D)**		When intravenous UFH is preferred (e.g. renal dysfunction), it can be used either with initial intravenous therapy followed by adjusted-dose subcutaneous UFH given every 12 h or q 12 h adjusted subcutaneous UFH alone. With subcutaneous therapy, UFH doses should be adjusted to prolong a midinterval (6 h post-injection) activated partial thromboplastin time into the therapeutic range **(no grade)**
Managing of life threatening PE				
Intravenous UFH can be considered in the initial treatment of PE and in situations in which thrombolysis may be necessary **(no grade)**	Thrombolytic therapy should only be considered in limb-threatening DVT or massive PE **(III-C)**	Collapsed shocked patients should be assessed by a team of experienced clinicians, including the on-call consultant obstetrician, who should decide on an individual basis whether a woman receives intravenous UFH, thrombolytic therapy or thoracotomy and surgical embolectomy. Management should involve a multidisciplinary team including senior physicians, obstetricians and radiologists **(no grade)** Intravenous UFH is the preferred, initial treatment in massive PE with cardiovascular compromise **(B)** The on-call medical team should be contacted immediately. An urgent portable echocardiogram or CT pulmonary angiogram within 1 h of presentation should be arranged. If massive PE is confirmed or, in extreme circumstances prior to confirmation, immediate thrombolysis should be considered **(no grade)**	Thrombolysis should only be considered in pregnancy for women with life or limb-threatening complications of acute VTE **(Consensus Level I)**	Thrombolytic therapy is best reserved for life-threatening VTE **(no grade)**
Graded compression stockings				
	Use of graded compression stockings can be considered for symptom relief in pregnant women with acute proximal DVT **(III-C)**	In the initial management of DVT, the leg should be elevated and a graduated elastic compression stocking applied to reduce edema. Mobilization with graduated compression stockings should be encouraged **(B)** Following a DVT, graduated compression stockings should be worn on the affected leg to reduce pain and swelling. Clinicians should be aware that the role of compression stockings in the prevention of post-thrombotic syndrome is unclear **(B)**	All women with confirmed DVT should wear a below-knee class 2 (30-40 mmHg) compression stocking for two years **(Consensus Level I)**	In patients with acute symptomatic DVT of the leg, suggest the use of compression stockings **(Grade 2B)** [24]
Treatment duration				
	Following acute VTE, therapeutic anticoagulation is recommended for a minimum of 3 months **(I-A)**	Treatment with therapeutic doses of subcutaneous LMWH should be employed during the remainder of the pregnancy **(B)** Therapeutic anticoagulant therapy should be continued for the duration of the pregnancy and for at least 6 weeks postnatally and until at least 3 months of treatment has been given in total. Before discontinuing treatment, the continuing risk of thrombosis should be assessed **(C)**	Anticoagulant therapy in pregnant women with acute proximal DVT and/or PE should be continued until at least 6 weeks postpartum or longer, if necessary, to complete a minimum total treatment period of 6 months **(Consensus Level I)** A shorter total duration of therapy (6–8 weeks) may be appropriate in women with isolated distal DVT, with consideration given to prophylactic dose LMWH for the remainder of the pregnancy **(Consensus Level I)**	For pregnant women with acute VTE suggest that anticoagulants should be continued for at least 6 weeks postpartum for a minimum duration of therapy of 3 months) in comparison with shorter durations of treatment **(Grade 2C)**
Insertion of IVC filter				
Women with recurrent VTE despite therapeutic anticoagulation may be candidates for IVC filter placement **(no grade)**	IVC filters should only be used in pregnant women with acute PE or DVT and contraindications to anticoagulation **(III-C)**	Consideration should be given to the use of a temporary IVC filter in women with proven DVT who have recurrent PE despite adequate anticoagulation and in the peripartum period for patients with iliac vein DVT to reduce the risk of PE **(D)**	Insertion of a temporary IVC filter should only be considered in pregnant patients with recent acute venous thrombosis in whom therapeutic anticoagulation is contraindicated because of a high risk of bleeding or who have objectively confirmed recurrent VTE despite therapeutic anticoagulation **(Consensus Level I)**	Insertion of temporary IVC filters is best restricted to women with proven DVT who have recurrent PE despite adequate anticoagulation (**no grade)**
Laboratory investigations				
	For pregnant women initiated on LMWH, a baseline platelet count should be done and repeated a week later to screen for HIT **(III-C)**	Routine measurement of peak anti-Xa activity for patients on LMWH for treatment of acute VTE in pregnancy or postpartum is not recommended except in women at extremes of body weight (less than 50 kg and 90 kg or more) or with other complicating factors (for example with renal impairment or recurrent VTE) putting them at high risk **(C)** Routine platelet count monitoring should not be carried out (unless UFH has been given) **(D)**	There is insufficient evidence to recommend monitoring of anti-Xa levels to guide dosing in women on therapeutic dose LMWH **(Consensus Level I)**	Suggest no routine monitoring of LMWH dosing with anti-Xa LMWH **(no grade)** For patients receiving heparin in whom clinicians consider the risk of HIT to be <1 %, suggest that platelet counts not be monitored **(Grade 2C)** (estimated incidence of HIT in obstetrics patients, <0.1 %)^25^

Please see individual references for grading criteria

**Table 2C:** Guideline summary—prevention of first and recurrent pregnancy-associated VTE

Pre-pregnancy counselling
SOGC: Individualized risk assessment for VTE before pregnancy, once pregnancy achieved, and as new clinical situations arise; consider patients’ values and preferences **(III-B)** RCOG: Documented risk assessment for VTE in early pregnancy or pre-pregnancy; repeat if hospitalization or other intercurrent problems; as well as intrapartum or postpartum **(C)** Discuss risk of VTE and reasons for individuals recommendations with patients **(no grade)**
Thrombophilia testing
ACOG: Test for antiphospholipid antibodies **(B)** and inherited thrombophilias **(C)** when history of thrombosis SOGC: No routine screening for inherited thrombophilias in women with first VTE in pregnancy **(III-C)** Test for deficiencies of protein S, protein C and antithrombin following VTE in pregnancy if family history of those thrombophilias or thrombosis at unusual site **(III-C)** Test for antiphospholipid antibodies if could affect duration of anticoagulation **(III-C)** RCOG: Before testing, counsel women on implications of testing for themselves and family; interpretation of results should be conducted by clinician with specific expertise **(no grade)** Test for antithrombin deficiency if family history of VTE and either antithrombin deficiency or no specific thrombophilia detected (**no grade)** Test for antiphospholipid antibodies if unprovoked VTE **(no grade)** Consider thrombophilia testing even if no personal history or risk factors for VTE in the presence of history of unprovoked or estrogen-provoked VTE in first-degree relative when aged <50 years; more informative if relative has known thrombophilia **(D)**
Heterozygosity for factor V leiden or prothrombin gene mutation
Antepartum ACOG: Clinical surveillance or prophylactic LMWH or UFH **(no grade)** SOGC: Clinical surveillance **(no grade)** RCOG: Consider prophylaxis if ≥3 other risk factors^C^; consider prophylaxis from 28 weeks if 2 other risk factors^**C**^ **(D)** ACCP: Clinical surveillance **(Grade 2C)** Postpartum ACOG: Clinical surveillance or anticoagulation if risk factors^**A**^ **(no grade)** SOGC: Clinical surveillance or 6 weeks **(II-3B)** prophylaxis if any ≥1 additional risk factors with absolute risk of VTE <1 % in isolation^B^ **(II-2B to III-B)** ROCG: Consider prophylaxis for at least 10 days if 1 other risk factor^C^; consider extending to 6 weeks if family history of VTE **(D)** AACP: Clinical surveillance if no family history; 6 weeks prophylaxis with prophylactic- or intermediate-dose LMWH or vitamin K antagonists targeted at INR 2.0 to 3.0 if family history **(Grade 2C)**
Protein C or S deficiency
Antepartum ACOG: Clinical surveillance or prophylactic LMWH or UFH **(no grade)** SOGC: Clinical surveillance **(no grade)** RCOG: Refer to local expert; consider antenatal LMWH prophylaxis **(D)** ACCP: Clinical surveillance **(Grade 2C)** Postpartum ACOG: Clinical surveillance or anticoagulation if risk factors^A^ **(no grade)** SOGC: Clinical surveillance or 6 weeks **(II-3B)** prophylaxis if any ≥1 additional risk factors with absolute risk of VTE <1 % in isolation^B^ **(II-2B to III-B)** RCOG: 6 weeks postnatal prophylaxis **(D)** ACCP: Clinical surveillance if no family history; 6 weeks postpartum prophylaxis with prophylactic- or intermediate-dose LMWH if family history **(Grade 2C)**
Compound heterozygosity
Antepartum ACOG: Prophylactic LMWH or UFH **(no grade)** SOGC: Prophylactic LMWH **(III-B)** RCOG: Refer to local expert; consider antenatal prophylaxis **(D)** ACCP: Clinical surveillance **(Grade 2C)** Postpartum ACOG: Anticoagulation SOGC: 6 weeks **(II-3B)** postpartum prophylactic LMWH **(II-2B)** RCOG: 6 weeks postnatal prophylaxis **(D)** ACCP: Clinical surveillance if no family history; 6 weeks prophylaxis with prophylactic- or intermediate-dose LMWH or vitamin K antagonists targeted at INR 2.0–3.0 if family history **(Grade 2C)**
Homozygosity for factor V leiden or prothrombin gene mutation
Antepartum ACOG: Prophylactic LMWH or UFH **(no grade)** SOGC: Prophylactic LMWH (**II-2A** for factor V Leiden; **III-B** for prothrombin gene mutation) RCOG: Refer to local expert; consider antenatal prophylaxis **(D)** AACP: Clinical vigilance if no family history **(Grade 2B)**; prophylactic or intermediate-dose LMWH if family history **(Grade 2B)** Postpartum ACOG: Anticoagulation **(no grade)** SOGC: 6 weeks **(II-3B)** postpartum prophylactic LMWH **(II-2B)** RCOG: 6 weeks postnatal prophylaxis **(D)** ACCP: 6 weeks postpartum prophylaxis with prophylactic- or intermediate-dose LMWH or vitamin K antagonists targeted at INR 2.0–3.0 **(Grade 2B)**
Antithrombin deficiency
Antepartum ACOG: Prophylactic LMWH or UFH **(no grade)** SOGC: Prophylactic LMWH **(III-B)** RCOG: Refer to local expert; consider antenatal prophylaxis **(D)** ACCP: Clinical surveillance **(Grade 2C)** Postpartum ACOG: Anticoagulation SOGC: 6 weeks **(II-3B)** postpartum prophylactic LMWH **(II-2B)** RCOG: 6 weeks postnatal prophylaxis **(D)** ACCP: Clinical surveillance if no family history; 6 weeks prophylaxis with prophylactic- or intermediate-dose LMWH or vitamin K antagonists targeted at INR 2.0–3.0 if family history **(Grade 2C)**
Antiphospholipid antibody
Antepartum ACOG: Clinical surveillance or prophylactic heparin **(C)** SOGC: Intermediate or therapeutic dose LMWH **(III-B)** RCOG: Consider antenatal prophylaxis throughout pregnancy if ≥3 other risk factors^C^; consider prophylaxis from 28 weeks if 2 other risk factors^**C**^ **(D)** Postpartum ACOG: 6 weeks postpartum anticoagulation **(C)** SOGC: 6 weeks **(II-3B)** postpartum prophylactic LMWH **(II-2B)** RCOG: 6 weeks postnatal prophylaxis **(D)**
Clinical risk factors
Antepartum SOGC: Assisted reproduction—no routine prophylaxis for ovulation induction **(III-C)**; if severe ovarian hyperstimulation syndrome, prophylaxis with ≥8–12 weeks LMWH after resolution **(III-B)**; consider prophylaxis with LMWH if increased risk of VTE **(III-B)**; non-obstetrical surgery during pregnancy—prophylactic LMWH with duration dependent upon procedure and patient **(III-B)**; strict bed rest—prophylactic LMWH if ≥ 7 days and BMI >25 kg/m^2^ at first antenatal visit **(II-2B)**; Multiple clinical risk factors—Prophylactic LMWH if absolute estimated VTE risk >1 %, especially if bed rest **(II-2B)** RCOG: Hospitalization—offer prophylaxis with LMWH unless contraindication such as risk of labor or active bleeding **(D)**; Hospitalization for hyperemesis—prophylaxis with LMWH and discontinue upon resolution **(C)**; assisted reproduction—if ovarian hyperstimulation syndrome, prophylaxis with LMWH in first trimester **(C)**; if IVF pregnancy and 3 other risk factors^**C**^, prophylaxis with LMWH in the first trimester **(C);** Women with ≥4 clinical risk factors^C^ should be considered for antennal LMWH throughout pregnancy; if 3 risk factorsc, start prophylaxis at 28 weeks **(D)** ACCP: Assisted reproduction—no routine thromboprophylaxis **(Grade 1B)**; if severe ovarian hyperstimulation syndrome, 3 mo prophylactic LMWH after resolution **(Grade 2C)** Postpartum SOGC: Strict bed rest—prophylactic LMWH if ≥7 days prior to delivery **(II-2B)**; blood loss—prophylactic LMWH if >1 L peripartum or postpartum or requiring blood product replacement and concurrent postpartum surgery **(II-2B)**; peripartum/postpartum infection—prophylactic LMWH **(II-B)**; multiple clinical risk factors—6 weeks prophylaxis if risk factors ongoing or persistent **(II-3B)** and while hospitalized or up to 2 weeks if transient **(III-C)** if any ≥2 risk factors with absolute risk of VTE <1 % in isolation^B^ **(II-2B to III-B)** or ≥3 risk factors with absolute risk of VTE of <1 %^**D**^ **(II-2B to III-B)** RCOG: Prophylactic LMWH dosed by weight for 10 days after delivery **(D)** if class 3 obesity (BMI ≥ 40 kg/m^2^) **(D)** or ≥ 2 risk factors^**C**^ **(B)**
Prevention of recurrent VTE
Single prior episode of VTE, not receiving long-term anticoagulation
Known Thrombophilia
Heterozygosity for Factor V Leiden or Prothrombin Gene Mutation
Antepartum ACOG: Prophylactic- or intermediate-dose LMWH or UFH or clinical surveillance **(no grade)** SOGC: Prophylactic LMWH (**I-A** if previous event provoked, **II-2A** if previous event unprovoked or related to oral contraceptives or pregnancy) RCOG: Prophylactic LMWH throughout pregnancy **(C)** ACCP: Low risk of recurrent VTE—(single episode associated with a transient risk factor not related to pregnancy or use of estrogen) clinical vigilance **(Grade 2C)**; moderate to high risk of recurrent VTE—(single unprovoked VTE, pregnancy-or estrogen-related VTE, or multiple prior unprovoked VTE) not receiving long-term anticoagulation, prophylactic-or intermediate-dose LMWH **(Grade 2C)** Postpartum ACOG: Anticoagulation or intermediate dose LMWH or UFH **(no grade)** SOGC: Postpartum 6 weeks **(II-3B)** prophylactic LMWH **(II-2A)** ROCG: ≥6 weeks thromboprophylaxis with LMWH or warfarin **(B)** ACCP: 6 weeks prophylactic-or intermediate-dose LMWH or vitamin K antagonists targeted at an INR 2.0–3.0 **(Grade 2B)**
Protein C or S deficiency
Antepartum ACOG: Prophylactic- or intermediate-dose LMWH or UFH or clinical surveillance **(no grade)** SOGC: Prophylactic LMWH (**I-A** if previous event provoked, **II-2A** if previous event unprovoked or related to oral contraceptives or pregnancy) RCOG: Prophylactic LMWH throughout pregnancy **(C)** ACCP: Low risk of recurrent VTE—(single episode associated with a transient risk factor not related to pregnancy or use of estrogen) clinical vigilance **(Grade 2C)**; moderate to high risk of recurrent VTE—(single unprovoked VTE, pregnancy-or estrogen-related VTE, or multiple prior unprovoked VTE) not receiving long-term anticoagulation, prophylactic-or intermediate-dose LMWH **(Grade 2C)** Postpartum ACOG: Anticoagulation or intermediate dose LMWH or UFH **(no grade)** SOGC: Postpartum 6 weeks **(II-3B)** prophylactic LMWH **(II-2A)** ROCG: ≥6 weeks thromboprophylaxis with LMWH or warfarin **(B)** ACCP: 6 weeks prophylactic-or intermediate-dose LMWH **(Grade 2B)**
Compound heterozygosity
Antepartum ACOG: Prophylactic-, intermediate-dose or adjusted-dose LMWH or UFH **(no grade)** SOGC: Intermediate- or therapeutic-dose LMWH **(III-B)** RCOG: Prophylactic LMWH **(D)** ACCP: Same as for heterozygosity for factor V Leiden or prothrombin gene mutation Postpartum ACOG: 6 weeks anticoagulation or intermediate- or adjusted-dose LMWH or UFH **(no grade)** SOGC: 6 weeks **(II-3B)** prophylactic LMWH **(II-2B)** RCOG: Prophylaxis with LMWH or warfarin for at least 6 weeks after delivery **(B)** ACCP: 6 weeks prophylactic-or intermediate-dose LMWH or vitamin K antagonists targeted at an INR 2.0 to 3.0 **(Grade 2B)**
Antithrombin deficiency
Antepartum ACOG: Prophylactic-, intermediate-dose or adjusted-dose LMWH or UFH **(no grade)** SOGC: Intermediate- or therapeutic-dose LMWH **(III-B)** RCOG: Management should be undertaken in collaboration with hematologist with expertise in thrombosis in pregnancy and consideration given to antenatal anti-Xa monitoring and the potential for antithrombin replacement at initiation of labor or prior to caesarean section; if anti-Xa levels measured, a test that does not use exogenous antithrombin should be used with a 4-h peak level target of 0.5–1.0 iu/ml (no grade); higher dose LMWH (50 or 75 % of treatment dose or weight-adjusted) **(D)** ACCP: Same as for heterozygosity for factor V Leiden or prothrombin gene mutation Postpartum ACOG: 6 weeks anticoagulation or intermediate- or adjusted-dose LMWH or UFH **(no grade)** SOGC: 6 weeks **(II-3B)** prophylactic LMWH **(II-2B)** RCOG: 6 weeks of prophylaxis with higher dose LMWH (50, 75 %, full treatment dose) or until returned to oral anticoagulant therapy **(B)** ACCP: 6 weeks prophylactic-or intermediate-dose LMWH or vitamin K antagonists targeted at an INR 2.0 to 3.0 **(Grade 2B)**
Antiphospholipid antibody
Antepartum ACOG: Prophylactic anticoagulation with heparin throughout pregnancy **(C)** SOGC: Intermediate or therapeutic dose LMWH **(III-B)** RCOG: Management should be undertaken in collaboration with a hematologist and/or rheumatologist with relevant expertise in women with antiphospholipid antibody syndrome. These women require specialist management by an expert in thrombosis and pregnancy; higher dose LMWH (either 50, 75 % or full treatment dose) **(D)** ACCP: Same as for heterozygosity for factor V Leiden or prothrombin gene mutation Postpartum ACOG: 6 weeks anticoagulation with heparin **(C)** SOGC: 6 weeks **(II-3B)** prophylactic LMWH **(II-2B)** RCOG: 6 weeks thromboprophylaxis with higher dose LMWH (either 50, 75 % or full treatment dose) or until returned to oral anticoagulant therapy **(D)** ACCP: 6 weeks prophylactic-or intermediate-dose LMWH or vitamin K antagonists targeted at an INR 2.0 to 3.0 **(Grade 2B)**
No known thrombophilia
Previous event associated with non-estrogen-related transient risk factor
Antepartum ACOG: Clinical surveillance**(no grade)** SOGC: Clinical surveillance **(II-2A)** RCOG: If original VTE provoked by major surgery from which they have recovered and no other risk factors, thromboprophylaxis with LMWH withheld until 28 weeks with close surveillance for the development of other risk factors; if additional risk factors, offer LMWH **(D);** if original VTE related to nonsurgical transient risk factor, offer LMWH throughout the antenatal period **(D)** ACCP: Low risk of recurrent VTE—(single episode associated with a transient risk factor not related to pregnancy or use of estrogen) clinical vigilance **(Grade 2C)** Postpartum ACOG: Anticoagulation (no grade) SOGC: 6 weeks **(II-3B)** prophylactic LMWH **(II-2A)** RCOG: Prophylaxis with LMWH or warfarin for at least 6 weeks after delivery **(B)** ACCP: 6 weeks prophylactic-or intermediate-dose LMWH or vitamin K antagonists targeted at an INR 2.0 to 3.0 (if not protein C or S deficient) **(Grade 2B)**
Previous event associated with pregnancy or estrogen-related
Antepartum ACOG: Prophylactic-dose LMWH or UFH **(no grade)** SOGC: Prophylactic-dose LMWH **(II-2A)** RCOG: Thromboprophylaxis with LMWH **(D)** AACP: Moderate to high risk of recurrent VTE—(single unprovoked VTE, pregnancy-or estrogen-related VTE, or multiple prior unprovoked VTE) not receiving long-term anticoagulation, prophylactic-or intermediate-dose LMWH **(Grade 2C)** Postpartum ACOG: Anticoagulation **(no grade)** SOGC: 6 weeks **(II-3B)** prophylactic LMWH **(II-2A)** RCOG: Prophylaxis with LMWH or warfarin for at least 6 weeks after delivery **(B)** ACCP: 6 weeks prophylactic-or intermediate-dose LMWH or vitamin K antagonists targeted at an INR 2.0 to 3.0 (if not protein C or S deficient) **(Grade 2B)**
Previous event unprovoked
Antepartum ACOG: Prophylactic-dose LMWH or UFH **(no grade)** SOGC: Prophylactic-dose LMWH **(II-2A)** RCOG: Thromboprophylaxis with LMWH **(D)** **AACP**: Moderate to high risk of recurrent VTE—(single unprovoked VTE, pregnancy-or estrogen-related VTE, or multiple prior unprovoked VTE) not receiving long-term anticoagulation, prophylactic-or intermediate-dose LMWH **(Grade 2C)** Postpartum ACOG: Anticoagulation **(no grade)** SOGC: 6 weeks **(II-3B)** prophylactic LMWH **(II-2A)** RCOG: Prophylaxis with LMWH or warfarin for at least 6 weeks after delivery **(B)** ACCP: 6 weeks prophylactic-or intermediate-dose LMWH or vitamin K antagonists targeted at an INR 2.0 to 3.0 (if not protein C or S deficient) **(Grade 2B)**
Two or more episodes, not receiving long-term anticoagulation
Antepartum ACOG: Prophylactic- or intermediate-dose LMWH or UFH **(no grade)** SOGC: As per single episode recommendations RCOG: These patients require specialist management by an expert in thrombosis and pregnancy; higher-dose LMWH (50–75 % of treatment dose or weight adjusted) **(D)** ACCP: Moderate to high risk of recurrent VTE—(single unprovoked VTE, pregnancy-or estrogen-related VTE, or multiple prior unprovoked VTE) not receiving long-term anticoagulation, prophylactic-or intermediate-dose LMWH **(Grade 2C)** Postpartum ACOG: 6 weeks anticoagulation or therapeutic-dose LMWH or UFH **(no grade)** SOGC: As per single episode recommendations RCOG: 6 weeks of prophylaxis with higher dose LMWH (50, 75 % or full treatment dose) **(D)** ACCP: 6 weeks prophylactic-or intermediate-dose LMWH or vitamin K antagonists targeted at an INR 2.0 to 3.0 (if not protein C or S deficient) **(Grade 2B)**
Two or more episodes, receiving long-term anticoagulation
Antepartum ACOG: Therapeutic-dose LMWH or UFH **(no grade)** SOGC: Therapeutic dose LMWH **(III-B)** RCOG: Women should be counselled about the risks of vitamin K antagonists to the fetus and advised to stop these medications and change to LMWH as soon as pregnancy is confirmed (ideally within 2 weeks of the missed period and before the 6th week of pregnancy) (no grade). Thromboprophylaxis with higher dose LMWH (either 50, 75 % or full treatment dose) **(D)** ACCP: If taking long-term vitamin K antagonists and candidates for LMWH substitution, suggest performing frequent pregnancy tests and substituting LMWH for vitamin K antagonists only when pregnancy achieved **(2C) [**Remark: women who place little value on avoiding the risks, inconvenience and costs of LMWH therapy of uncertain duration while awaiting pregnancy and a high value on minimizing the risks of miscarriage associated with vitamin K antagonists are likely to choose LMWH while attempting pregnancy]. Suggest adjusted-dose or therapeutic dose of LMWH **(Grade 2C)** Postpartum ACOG: Resumption of long-term anticoagulation **(no grade)** SOGC: Resumption of long-term anticoagulation (**no grade)** RCOG: Thromboprophylaxis with higher dose LMWH (either 50, 75 % or full treatment dose) for 6 weeks or until returned to oral anticoagulant therapy after delivery **(D).** Warfarin can be restarted in women receiving long-term anticoagulation with this agent when the risk of bleeding is reduced; usually 5–7 days after delivery **(no grade)** ACCP: Suggest resumption of long-term anticoagulants, rather than prophylactic LMWH **(Grade 2C)**
Prevention of VTE associated with caesarean section
ACOG: Placement of pneumatic compression devices before caesarean delivery if not already receiving thromboprophylaxis **(C)** SOGC: Following emergency caesarean section—if risk factors each with an absolute risk <1 % risk of VTE in isolation (other than cesarean section)^**B**^, consider LMWH prophylaxis **(II-2B to III-B)**; following elective caesarean section—if ≥ 2 risk factors each with an absolute risk of VTE of <1 % (other than cesarean section)^**D**^, consider LMWH **(II-2B to III-B)**; 6 weeks postpartum prophylaxis if risk persistent **(II-3B)**, while risk factors ongoing, and during hospitalization; up to 2 weeks postpartum prophylaxis if risk transient **(III-C)** RCOG: Emergency caesarean section—10 day thromboprophylaxis with LMWH after delivery **(C)**; all other patients undergoing caesarean section—consider 10 days thromboprophylaxis with LMWH after delivery if additional risk factors^E^ **(C)** ACCP: No additional risk factors—no prophylaxis other than early mobilization **(Grade 1B)**; 1 major risk factor^F^ or ≥2 minor risk factors^G^ (1 minor if emergency caesarean section)—(risk of postpartum VTE of >3 %), suggest prophylactic LMWH (if contraindication to anticoagulation use mechanical prophylaxis (elastic stockings or intermittent pneumatic compression) while in hospital following delivery **(Grade 2B)** [Remark: the reduced risk of bleeding with mechanical prophylaxis should be weighed against inconvenience of elastic stockings and intermittent pneumatic compression]; very high risk with multiple additional risk factors persisting in puerperium—combine prophylactic LMWH with elastic stockings and/or intermittent pneumatic compression **(Grade 2C)**; selected high risk patients with significant risk factors persisting after delivery—up to 6 weeks extended prophylaxis after delivery following hospital discharg **(Grade 2C)**

Please see individual references for grading criteria

**Table 2D:** Guideline summary—anticoagulant management around the time of delivery

American College of Obstetricians and Gynecologists (ACOG) [17, 18]	Society of Obstetricians and Gynaecologists of Canada (SOGC) [19]	Royal College of Obstetricians and Gynaecologists (RCOG) [20, 21]	Australia/New Zealand [22]	American College of Chest Physicians (ACCP) [23]
Antepartum: general guidance				
	Women receiving prophylactic, intermediate-dose, or therapeutic anticoagulation should have a discussion about options for analgesia/anaesthesia prior to delivery **(III-B)**	Women on LMWH for treatment of VTE should be advised that once she is in established labour or thinks she is in labour, she should not inject any further heparin **(no grade)** LMWH maintenance therapy should be discontinued 24 h prior to planned delivery (induction of labour or caesarean section) **(D)**		Delivery options in women using anticoagulants are best considered by a multidisciplinary team. Several options are possible, including spontaneous labour and delivery, induction of labor, and elective caesarean section. The plan for delivery should take into account obstetric, hematologic, and anesthetic considerations **(no grade)** Women at very high risk for recurrent VTE (e.g. proximal DVT or PE close to the expected date of delivery may benefit by having a planned delivery by induction or caesarean section as appropriate, so that the duration of time without anticoagulation can be minimized **(no grade)**
Antepartum: conversion to UFH				
Women receiving therapeutic or prophylactic LMWH may be converted to unfractionated heparin in the last month of pregnancy or sooner if delivery appears imminent (Level C) or planned delivery with withholding of anticoagulants for 24 h **(no grade)**	Switching from prophylactic LMWH to UFH at term (37 weeks) may be considered to allow for more options with respect to labour analgesia **(III-L)**		Planned delivery with conversion to intravenous UFH in anticipation of delivery may be required to minimize the time off anticoagulants for women at the highest risk of recurrent VTE **(no grade)**	Women at highest risk of recurrent VTE (e.g. proximal DVT or PE within 2 weeks) can be switched to intravenous UFH prior to planned delivery, which is then discontinued 4–6 h prior to the expected time of delivery or epidural insertion **(no grade)**
Antepartum: Laboratory Investigations				
	A recent platelet count should be available on admission in labour or before caesarean delivery in women who have been, or are, on anticoagulants **(III-B)**			
Antepartum: timing of neuraxial blockade after last dose of LMWH/UFH				
It is recommended to withhold neuroaxial blockade for 10–12 h after the last prophylactic dose of LMWH or 24 h after the last therapeutic dose of LMWH **(Level C)**	Discontinue prophylactic or intermediate dose LMWH or UFH upon the onset of spontaneous labour or the day prior to a planned induction of labour or caesarean section **(II-3B)** For women on LMWH, neuroaxial anesthesia can be administered a minimum of 10-12 h after the last prophylactic dose (III-B) or at least 24 h after the last therapeutic dose **(III-B)** For women on UFH, neuroaxial anesthesia can be administered after no delay following a prophylactic dose (maximum 10,000 units/day) **(III-B)**, at least 4 h after stopping a therapeutic intravenous infusion (and when the activated partial thromboplastin time is normal) **(III-B)**, and 12 h or longer after the last therapeutic subcutaneous dose (and when the activated partial thromboplastin time is normal) **(III-B)** Neuroaxial anesthesia must be avoided in a women who is fully anticoagulated or in whom there is evidence of altered coagulation **(II-3A)**	Women taking LMWH should be advised that once she is in established labour or thinks she is in labour, she should not inject any further heparin **(no grade)** Where delivery is planned, LMWH therapy should be discontinued 24 h prior to delivery **(no grade)**. Regional anesthesia or analgesic techniques should not be taken until at least 24 h after the last dose of therapeutic LMWH **(no grade**) and at least 12 h after the last dose of prophylactic LMWH **(no grade)**	For prophylactic LMWH, a minimum of 12 h after LMWH dose is required prior to performance of neuroaxial blockade **(no grade)**. Wait at least 2 h following neuroaxial blockade or catheter removal before giving subsequent dose **(no grade)** For therapeutic LMWH, a minimum of 24 h after LMWH dose is required before performance of neuroaxial blockade **(no grade)** For prophylactic UFH (≤10,000 units daily), wait at least 6 h after last dose before performing neuroaxial blockade **(no grade)**. Wait at least 2 h after performing neuroaxial blockage or removing neuroaxial catheter before giving next dose **(no grade)** For therapeutic UFH, stop intravenous UFH 4–6 h prior to performing neuroaxial blockade and document a normal activated partial thromboplastin time (3–4 h after stopping infusion)**(no grade)**. Wait at least 1 h after performing neuroaxial blockage or removing neuroaxial catheter before restarting UFH **(no grade)**	For pregnant women receiving adjusted-dose LMWH and where delivery is planned, recommend discontinuation of LMWH at least 24 h prior to induction of labour or caesarean section (or expected time of neuroaxial anesthesia) rather than continuing LMWH up until the time of delivery **(Grade 1B)** If spontaneous labour occurs in women receiving anticoagulation, neuroaxial anesthesia should not be used. Where the level of anticoagulation is uncertain and where laboratory support allows for rapid assessment of heparin levels, then testing can be considered to guide anesthetic and surgical management **(no grade)**
Antepartum: insertion of IVC filter				
Women with DVT within 2–4 weeks before delivery may be candidates for placement of a retrievable IVC filter **(no grade)**		Consideration should be given to the use of a temporary IVC filter in the perinatal period for women with iliac vein DVT to reduce the risk of PE **(no grade)**		Women at highest risk of recurrent VTE (e.g. proximal DVT or PE within 2 weeks) can have a temporary IVC filter inserted prior to planned delivery and removed postpartum **(no grade)**
Postpartum: general advice—including management of women at high risk of hemorrhage				
When reinstitution of anticoagulant therapy is planned postpartum, pneumatic compression devices should be left in place until the patient is ambulatory and until anticoagulation is restarted **(Level C)**		In women receiving therapeutic doses of LMWH, wound drain (abdominal and rectus sheath) should be considered at caesarean section and the skin incision should be closed with staples or interrupted sutures to allow for drainage of any hematoma **(no grade)** Any women who is considered to be at high risk of hemorrhage and in whom continued heparin treatment is considered essential should be manage with intravenous UFH until the risk factors for hemorrhage have resolved **(no grade)**		
Postpartum: timing of resumption of anticoagulant therapy				
Resumption of anticoagulation therapy no sooner than 4–6 h after vaginal delivery or 6–12 h after caesarean delivery is a reasonable approach to minimize bleeding complications **(Level B)**	Removal of a neuraxial catheter left in situ postpartum should only be undertaken 4, 10, 12, or 24 h following the administration of prophylactic dose UFH (maximum 10,000 units daily), prophylactic dose LMWH **(single daily dose**), or therapeutic dose LMWH, respectively; or in the case of therapeutic dose UFH when the activated partial thromboplastin time is normal **(II-3B)** Prophylactic LMWH (single daily dose) may be started/restarted 4 h after neuroaxial catheter removal, provided there is full neurologic recovery and no evidence of activated bleeding or coagulopathy **(III-B)** Therapeutic LMWH may be started/restarted at least 24 h after a single injection neuroaxial block and a minimum of 4 h after neuroaxial catheter removal, provided there is full neurologic recovery and no evidence of activated bleeding or coagulopathy **(III-B)** Subcutaneous UFH may be started/restarted at least 1 h after a single injection neuroaxial block, provided there is full neurologic recovery and no evidence of activated bleeding or coagulopathy **(III-B)** Concomitant antiplatelet agents (acetylsalicylic acid or nonsteroidal anti-inflammatory drugs) should not be administered concomitantly with heparin if a neuroaxial catheter is left in situ postpartum **(III-D)** Women on therapeutic anticoagulation who have received neuroaxial anesthesia should be monitored closely for the development of spinal hematoma **(III-B)**	The first prophylactic dose of LMWH should be given as soon as possible after delivery provided there is no postpartum hemorrhage and regional anesthesia has not been used **(no grade)** LMWH should not be given for 4 h after the use of spinal anesthesia or after the epidural catheter has been removed and the epidural catheter should not be removed within 12 h of the most recent injection **(D)** Women should be offered a choice of LMMH or vitamin K antagonists for postnatal therapy after discussion about the need for regular blood tests for monitoring warfarin, particularly during the first 10 days of treatment **(no grade)** Initiation of warfarin should be avoided until at least the 5th day and for longer in women at increased risk of postpartum hemorrhage **(no grade)**		

Please see individual references for grading criteria

*ACOG* American College of Obstetricians and Gynecologists, *ACCP* American College of Chest Physicians, *ART* Assisted reproductive technology, *DVT* deep vein thrombosis, *HIT* heparin-induced thrombocytopenia, *IVF* In vitro fertilization, *IVC* inferior vena cava, *LMWH* low molecular weight heparin, *RCOG* Royal College of Obstetricians and Gynaecologists, *SOGC* Society of Obstetricians and Gynecologists of Canada, *PE* pulmonary embolism, *UFH* unfractionated heparin, *VTE* venous thromboembolism

In making recommendations regarding the need for prophylaxis, the panel used a risk threshold of 3 % and greater for antepartum prophylaxis and 3 % and greater for postpartum prophylaxis. For risk factors for which only case control data are available, a relative risk of at least 30-fold antepartum and 60-fold postpartum are required to reach our thresholds, assuming antepartum and postpartum baseline risks of 0.1 and 0.05 %, respectively [26]. The prophylaxis thresholds above were determined by the majority result of an anonymous vote of the authors. It is important to note that there was inconsistency between the authors in their risk threshold for recommending prophylaxis. For antepartum prophylaxis, three chose 3 % or greater, one 5 % or greater, and one 1 % or greater. For postpartum prophylaxis, four selected a threshold of 3 % or greater and one chose 1 % or greater. The variability in risk thresholds is not surprising given the limitations of the available evidence, as well as the competing benefits and drawbacks of prophylaxis. The panel would emphasize that changes in the antepartum threshold to 5 or 1 % and to the postpartum threshold to 1 % would markedly change the recommendations that follow. When making recommendations, the panel also took into account the estimated risks of major bleeding with prophylactic LMWH (antepartum: 0; 95 % CI 0–0.6 % and postpartum: 0.3 %; 95 % CI 0–1.0 %) [26]; the variability in risk estimates reported in the literature, the 95 % confidence intervals around the risk estimates, and the strengths or weaknesses of relevant study methodology in addition to the above threshold limits.

## Guidance

1. What are the risks of anticoagulant use during pregnancy?

During pregnancy, the risks posed to the fetus by anticoagulant therapy, as well as maternal efficacy and safety must be considered. Vitamin K antagonists cross the placenta and have the potential to cause teratogenicity as well as pregnancy loss, fetal bleeding, and neurodevelopmental deficits [27–34]. Discontinuation of vitamin K antagonists prior to the 6th week of gestation essentially eliminates the risk of warfarin embryopathy [29, 30, 32]. Pregnant women were excluded from participating in clinical trials evaluating the oral direct thrombin and factor Xa inhibitors (e.g. dabigatran, rivaroxaban, apixaban, edoxaban). These agents are likely to cross the placenta and their human reproductive risks are unknown [35–38]. Fondaparinux appears to cross the placenta in small quantities [39]. Reports of the successful use of fondaparinux in pregnant woman have been published [39–46] but it is important to recognize that many of these involve second trimester or later exposure.

Unfractionated heparin (UFH), low molecular weight heparin (LMWH) and danaparoid (a heparinoid) do not cross the placenta and are safe for the fetus [47–55]. Although UFH can be used during pregnancy for both prevention and treatment of thromboembolism, LMWH has a better safety profile than UFH [56, 57] and the incidence of bleeding and other complications (e.g. heparin induced thrombocytopenia [HIT], and heparin-associated osteoporosis) are lower in pregnant women receiving LMWH than with UFH [58–69]. LMWHs are eliminated primarily by renal excretion and may accumulate in patients with significant renal dysfunction. In the non-pregnant population, it has been suggested that therapeutic dose LMWH not be used in patients with significant renal impairment (e.g. a glomerular filtration rate (GFR) of less than 30 mL/min), although it is recognized that accumulation in patients with renal impairment may differ between the various LMWHs [70].

As outlined in Table 2A, there is clear consensus amongst the reviewed guideline documents that, in general, LMWH is the preferred anticoagulant for the management and treatment of VTE in pregnancy [17–25].

### **Guidance Statement**

- *Physicians should counsel women receiving long-term therapy with vitamin K antagonists and the oral direct-acting anticoagulants about the fetal risks of these medications before pregnancy occurs.*
- *LMWH is the drug of choice for treatment and prevention of VTE in pregnancy, except in patients with HIT, a history of HIT, or significant renal dysfunction. UFH is preferred in patients with significant renal dysfunction.*
- *For women taking vitamin K antagonists, two options are available to reduce the risk of warfarin embryopathy. The first is to advise women to perform frequent pregnancy tests and substitute LMWH for warfarin once pregnancy is achieved and before 6 weeks gestation. Alternatively, LMWH or UFH can be substituted for vitamin K antagonists before conception is attempted. Although the latter approach minimizes the risks of early miscarriage associated with vitamin K antagonist therapy, it lengthens the duration of exposure to LMWH or UFH and, therefore, is costly and exposes the patient to a greater burden of treatment associated with the use of injectable heparin therapy. Since warfarin embryopathy is unlikely to result from warfarin exposure before 6 weeks, the first option is usually favored by guidelines. Although the management of women who are receiving long-term therapy with oral direct thrombin and factor Xa inhibitors and attempting to conceive remains controversial, it has been suggested that these women should be converted to a coumarin or LMWH before conception is attempted; failing that, the switch should be made as soon as pregnancy is confirmed.*
- *In pregnant women with severe cutaneous allergies to UFH or LMWH or with HIT or a history of HIT, danaparoid or fondaparinux (if danaparoid not available) may be used. Appropriate dosage and management of these anticoagulants around the time of delivery should be discussed with a hematologist or thrombosis specialist.*

2. What are the risks of anticoagulation in breastfeeding women?

Neither warfarin, the most commonly used vitamin K antagonist in North America and the United Kingdom nor acenocoumarol, which is commonly used in Europe, is detected in breast milk and neither medication induces an anticoagulant effect in the breast-fed infant when nursing mothers consume the drug [71–74]. Phenprocoumon, another vitamin K antagonist with a long half-life, is also widely used outside of North America. This agent is more lipophilic than warfarin and acenocoumarol and so can be excreted into breast milk, although since it is highly protein-bound, the amounts detected are small [75, 76]. Small amounts of LMWH have been detected in the breast milk of women receiving this medication [77]; however, given the very low bioavailability of heparin when ingested orally [78], there is unlikely to be any clinically relevant effect on the nursing infant. All of the guidelines that address the issue of anticoagulant use during breastfeeding agree that warfarin, LMWH, and UFH are safe to use in this setting (see Table 2A) [17, 20–22]. Phencoumaron should be reserved for women who are unstable on short-acting acenocoumarol in countries where warfarin is not available [76].

According to the manufacturer’s prescribing information, fondaparinux was excreted in the milk of lactating rats [79]. There are no published data on the excretion of fondaparinux into human milk and the effects on the nursing infant are unknown. The manufacturer recommends that caution be used when administering fondaparinux to breastfeeding women [79]. That said, significant absorption by the nursing infant would be unlikely as orally ingested heparins have low bioavailability [78].

There are no clinical data on the effect of maternally ingested oral direct thrombin and factor Xa inhibitors on breastfed infants. The manufacturers of these agents all recommend against using these medications in breastfeeding women [35–37].

### **Guidance Statement**

- *UFH, LMWH, warfarin and acenocoumarol are safe for the breast-fed infant when administered to the nursing mother.*
- *The oral direct thrombin and factor Xa inhibitors should not be used while breastfeeding.*

3. How is venous thromboembolism during pregnancy treated?

The guideline recommendations for management of acute VTE during pregnancy are summarized in Table 2B. There have been no large studies examining the safety and efficacy of outpatient treatment of VTE diagnosed during pregnancy. Data from the non-pregnant population suggest that outpatient DVT treatment is not associated with an increase in mortality, recurrent VTE, or major bleeding [24]. In non-pregnant patients with acute DVT, outpatient treatment is recommended as long as the patient feels well enough to be treated at home (e.g. does not have severe leg symptoms or comorbidity) and has well-maintained living conditions, strong support from family or friends, telephone access, and the ability to quickly return to hospital if conditions deteriorate [24]. The safety of treating PE at home, even in the non-pregnant population, is uncertain. Prediction rules have been developed for identifying non-pregnant patients with acute PE who might be suitable for outpatient treatment because they are at low risk of serious complications [24, 80].

The results of large trials in non-pregnant patients demonstrating that LMWHs are at least as safe and effective as UFH for the acute treatment of VTE [81, 82] and as vitamin K antagonists for the prevention of recurrent VTE [83, 84], as well as data from subsequent observational studies in pregnant women, support the use of LMWH for treatment of VTE in this patient population [60, 61, 85, 86].

There are no large trials examining the optimal dose of anticoagulants for treatment of acute VTE during pregnancy. Some pharmacokinetic studies suggest that increases in GFR and in patient weight (and, hence, LMWH volume of distribution) that occur during pregnancy may lead to lower LMWH levels and that the dose of LMWH should be adjusted over the course of pregnancy to maintain “therapeutic” anti-Xa LMWH levels [87, 88], or according to changes in weight [89]. However, other researchers have demonstrated that few women require dose-adjustment when therapeutic doses of LMWH are used [90–94]. Some recommend a twice-daily LMWH dosing schedule during pregnancy to compensate for increases renal clearance of this medication that occur in the second trimester. In non-pregnant patients, once daily LMWH is as safe and effective as twice daily LMWH when used to treat acute VTE [95]. Observational studies in pregnant women with acute VTE have not demonstrated any increase in the risk of recurrence with a once-daily regimen compared with twice-daily schedules [85, 86] and many clinicians use once-daily therapy to simplify administration and enhance compliance.

There are issues with reliability of anti-Xa LMWH tests [96, 97] and these assays are costly. In the absence of robust data demonstrating that there is an optimal “therapeutic anti-Xa LMWH range” and that dose-adjustments increase the safety or efficacy of LMWH therapy, current guidelines do not mandate routine monitoring of LMWH with anti-Xa levels [21–23]. Anti-Xa monitoring may be helpful to ensure appropriate anticoagulant effect in patients with renal impairment and in those at the extremes of body weight [21].

Regimens in which the intensity of LMWH is reduced later during the course of therapy to an intermediate dose regimen [98] or 75 % of a full treatment dose [84] have been used successfully in cancer patients. A recent systematic review that identified four studies in which pregnant women with symptomatic VTE were transitioned from full-dose anticoagulation to intermediate-dose LMWH (less than 75 % of a full treatment dose but greater than prophylactic dose) within 6 weeks of VTE diagnosis, reported a low risk of VTE recurrence (one of 152 patients) during intermediate-dose LMWH therapy; however, the number of patients with PE was small (four) and at least one of the included studies enrolled patients with isolated calf vein thrombosis, which could lead to an overestimation of the positive effect [99]. Some guidelines suggest a dose-reduction strategy for pregnant women at risk of anticoagulant-related bleeding and heparin-induced osteoporosis [23] and in those with isolated calf vein thrombosis [22]. That said, a survey of members of the North American Society of Obstetric Medicine and Thrombosis Canada found that only one-quarter of respondents utilized this strategy in their patients [100].

The risk of HIT in pregnant women treated with LMWH alone is low (less than 0.1 %) [59]; it is higher in pregnant women who have received UFH. Several guidelines suggest that routine platelet count monitoring for detection of HIT is not required in pregnant women treated exclusively with LMWH [21, 25].

Intravenous UFH is preferred when rapid reversal of anticoagulation may be required (i.e. in situations in which urgent delivery or surgery may be necessary) and in patients in whom thrombolysis may be considered (e.g. high risk or massive PE) [24, 80]. UFH should be used in preference to LMWH to treat acute VTE in patients with GFR of less than 30 mL/min [80]. When UFH is preferred, it can be given intravenously or subcutaneously every 12 h in doses adjusted to prolong a mid-interval (6 h post-injection) activated partial thromboplastin time (aPTT) into therapeutic range [101], although it is recognized that aPTT monitoring is less reliable in pregnancy [102].

Concerns about the use of thrombolytic therapy during pregnancy center on its maternal effects (major hemorrhage), as well as those on the placenta (i.e. premature labor, placental abruption, fetal demise), as transplacental passage of tissue plasminogen activator and streptokinase is minimal [103]. There have been several reports of successful thrombolysis in pregnancy with no harm to the fetus; however, the number of cases is small and most cases involved streptokinase [104–107]. Therefore, there is agreement amongst available guidelines that the use of thrombolytic therapy in pregnancy is best reserved for limb or life-threatening maternal thromboembolism (e.g. PE with refractory cardiorespiratory compromise) [21–23, 80].

There is limited experience with inferior vena caval filters during pregnancy and serious complications, including filter fracture, filter migration, failed retrieval of temporary devices, and inferior vena caval perforation, have been reported [108–112]. Current guidelines recommend insertion of temporary inferior vena caval filters in pregnant women with acute VTE and contraindications to anticoagulant therapy [18, 22] or recurrent VTE despite therapeutic anticoagulation [17, 21–23]. An alternate strategy involving anticoagulant dose-escalation may also be appropriate for managing the latter situation, based on favorable (but limited) data in cancer patients, in which recurrent VTE despite anticoagulant therapy is treated by increasing the dose of LMWH by approximately 25 % or to therapeutic levels in those receiving lower doses [113, 114].

There are conflicting data concerning the long-term effectiveness of graduated compression stockings to prevent post-thrombotic syndrome. On the basis of two positive open label randomized trials in the non-pregnant population [115, 116], several guidelines have suggested that graduated compression stockings be prescribed to reduce the likelihood of developing post-thrombotic syndrome [19, 22, 23]. However, a recent multicenter placebo-controlled trial that enrolled non-pregnant patients reported that these stockings neither prevented this complication nor reduced the risk of recurrent VTE [117]. In addition, although it is thought that graduated stockings may be useful for acute symptom relief, a subgroup analysis of this study suggests that, at least in the non-pregnant population, this may not be the case [118].

There have been no studies assessing optimal duration of anticoagulant therapy for treatment of pregnancy-related VTE. In non-pregnant patients with VTE, evidence supports a minimum treatment duration of 3 months [24]. Given the increased risk of VTE in pregnant women and following delivery, available guidelines suggest that anticoagulants be continued throughout pregnancy and the postpartum period, and for a minimum of 3 months [19, 21–25].

### **Guidance Statement**

- *Outpatient treatment of VTE can be considered in patients who are clinically stable and have good cardiorespiratory reserve, no major risk factors for bleeding and good social support with easy access to medical care. Hospitalization is indicated in patients who are hemodynamically unstable or do not have good social support and those who have extensive VTE, or maternal co-morbidities that limit their tolerance of recurrent VTE or increase their risk of major bleeding.*
- *LMWH is the preferred anticoagulant for most pregnant women with acute VTE. UFH should be used instead of LMWH in patients with GFR less than 30 mL/min. Intravenous UFH should be considered in patients who may require thrombolysis, surgery or urgent delivery.*
- *If LMWH is used for treatment of acute VTE in pregnancy, the same weight-adjusted dosing regimen as in the nonpregnant population should be utilized (Table *3).* Routine monitoring of LMWH dosing with anti-Xa LMWH is likely not required.*

**Table 3:** Accepted LMWH dosing regimens for treatment of pregnancy-related VTE

Initial treatment	Therapeutic (adjusted-dose) LMWH Dalteparin 200 units/kg once daily or 100 units/kg every 12 h Tinzaparin 175 units/kg once daily Enoxaparin 1 mg/kg every 12 h Nadroparin 86 units/kgevery 12 h or 171 units/kg once daily
Dose adjustments	No dose adjustment For patients at risk for bleeding or osteoporosis and for those with isolated distal DVT, consideration can be given to decreasing the dose to 75 % of full treatment dose after at least a month of therapy
- *Thrombolytic therapy should be reserved for pregnant women with PE associated with life-threatening cardiorespiratory compromise or limb-threatening DVT.*
- *Insertion of a temporary inferior vena caval filter should be considered in pregnant women with acute VTE and a contraindication to anticoagulant therapy.*
- *Anticoagulant therapy for treatment of VTE during pregnancy should be continued throughout pregnancy and for at least 6 weeks postpartum for a minimum duration of 3 months.*

4. How is pregnancy-associated VTE prevented?

Decisions regarding the use of prophylactic anticoagulation during pregnancy depend on the balance between the estimated risk of VTE and associated reduction in risk with prophylaxis, along with the burdens associated with anticoagulant therapy. The appropriate use of prophylaxis depends on identifying those at sufficiently high risk of VTE to benefit from this intervention. Risk factors to be considered include prior VTE, familial VTE history, the presence of a known thrombophilia, and clinical factors, including cesarean delivery, prolonged antepartum immobilization, increased body mass index (BMI), as well as significant pregnancy complications and medical comorbidities.

Prophylaxis during pregnancy typically involves long-term subcutaneous injections of LMWH. Although prophylactic LMWH is safe for the fetus [27, 29, 30, 49–51] and does not appear to appreciably increase the risk of adverse maternal outcomes [58, 59, 62–67]; it is expensive, inconvenient and uncomfortable to administer. Depending on local practice, prophylaxis with LMWH may also necessitate a planned delivery to permit epidural analgesia and women may perceive that it creates an undesirable “medicalization” of their pregnancy.

A Cochrane systematic review of thromboprophylaxis in pregnancy and the early postnatal period that examined 16 randomized trials involving 2592 women concluded that the current available information is insufficient to make firm recommendations for prophylaxis [119]. Current clinical guidelines are based on these small trials, additional observational studies and indirect evidence suggesting that LMWH substantially decreases the risk of VTE in a wide variety of clinical settings. As shown in Table 2C, there is incomplete agreement between the guidelines as to which patients should receive thrombosis prophylaxis and only a few guidelines (SOGC [19] and ACCP for postpartum prophylaxis [23]) explicitly provide information about the risk threshold used to determine whether or not patients should receive prophylaxis.

Given the competing potential drawbacks and benefits of prophylaxis, as well as the limitations of the available evidence, the decision to use or not use LMWH is likely to be value and preference sensitive. In addition to holding different attitudes toward the risk of recurrent thrombosis and about the burdens associated with the use of prophylaxis, women are also likely to place varying importance on minimizing medicalization of their pregnancy. All women, therefore, merit an individualized risk–benefit assessment of their need for prophylaxis and the opportunity to share in a decision making process about this intervention that takes into account their values and preferences.

If the decision is made to use antepartum prophylaxis, it should be initiated early in pregnancy as there is evidence of an increased risk of VTE during all three trimesters [120, 121]. Postpartum prophylaxis is less burdensome than antepartum prophylaxis as the duration of prophylaxis is shorter (i.e. 6 weeks) and an oral anticoagulant is available for those uncomfortable with subcutaneous injections (vitamin K antagonists, except for those with protein C or S deficiency who are at risk for developing warfarin-induced skin necrosis) [122–124].

The optimal prophylaxis strategy is unknown. Several LMWH dosing regimens have been used for prophylaxis of VTE during pregnancy (Table 4) [59, 62, 118, 125–133]. Although all of the studies evaluating these regimens reported low VTE rates, most were cohort studies and, therefore, lacked data from untreated controls. Some investigators have reported failures of prophylactic LMWH; however, it is unclear whether these represent true failures or were due to noncompliance with long-term subcutaneous injections [59, 60, 127, 134, 135]. Different dosing strategies have not been directly compared, although one randomized trial comparing higher doses of LMWH prophylaxis with usual fixed dose prophylaxis is ongoing (Highlow Randomized Controlled Trial: Comparison of Low and Intermediate Dose Low-molecular-weight Heparin to Prevent Recurrent Venous Thromboembolism in Pregnancy; NCT001828697).

**Table 4:** Suggested LMWH dosing regimens for prophylaxis against pregnancy-related VTE

Prophylactic LMWH^a^
Dalteparin 5000 units once daily
Tinzaparin 4500 units once daily or 75 units/kg once daily
Enoxaparin 40 mg once daily
Nadroparin 2850 units once daily
Intermediate-dose LMWH^a^
Dalteparin 5000 units twice daily or 10,000 units once daily
Tinzaparin 10,000 units once daily
Enoxaparin 40 mg twice daily or 80 mg once daily
LMWH adjusted to a peak anti-Xa level of 0.2–0.6 units/mL

^a^Higher doses may be used in with increased maternal weight

In hospitalized women, mechanical prophylaxis with elastic stockings and/or intermittent pneumatic compression is an alternative for those with contraindications to anticoagulant prophylaxis [23]; although there is limited evidence that these devices are less effective at prevention of VTE [136].

Duration of anticoagulant prophylaxis after delivery remains controversial. Available guidelines recommend 6 weeks of postpartum prophylaxis in patients with prior VTE and those with some thrombophilias (varies between guidelines) [17, 19, 21, 23]. However, there is minimal evidence to guide duration of prophylaxis in women with other clinical risk factors and recommendations vary. A shorter course of postpartum prophylaxis (until discharge or for one to 2 weeks post discharge) is often suggested for women with transient risk factors [19, 21, 23]. A recent study that used linked primary and secondary care data to assess VTE risk during specific postpartum periods reported that women with pre-eclampsia/eclampsia and acute systemic infection, obesity (body mass index or BMI ≥ 30 kg/m^2^), and cesarean delivery had elevated VTE risks up to 6 weeks postpartum; while VTE risk was increased only for the first 3 weeks after delivery in those with postpartum hemorrhage or preterm birth [137]. However, the absolute VTE risk during those time frames was low (less than 1 %).

All pregnant women at risk of VTE should be educated about the signs and symptoms of DVT and PE and the need to seek urgent medical attention should they develop. Objective testing is mandatory if symptoms suspicious of DVT or PE occur.

*Prevention of recurrent VTE*

The most important individual risk factor for pregnancy-associated VTE is a prior history of thrombosis [138]. The absolute risk of recurrent VTE during pregnancy in women not given antepartum prophylaxis remains controversial. In more recent studies, the reported incidence ranged from 2.4 % (95 % CI 0.2–6.9) in a prospective study of 125 pregnant women [139] to approximately 6 % in larger retrospective cohort studies [140, 141]. Differences in study population (later median gestational age at enrollment; inclusion of women with more than one prior episode of VTE in the retrospective studies), as well as failure to independently adjudicate recurrent events in the retrospective studies, may explain the higher risk of recurrence in the latter studies. However, the overall risk of antepartum recurrent VTE in both prospective and retrospective studies was less than 10 % and CI’s around the risk estimates of individual studies are overlapping.

Data regarding prognostic factors for recurrent VTE during pregnancy are inconsistent. Although a subgroup analysis of the prospective cohort study mentioned above found a lower risk of recurrence in women without thrombophilia who had a temporary risk factor (including oral contraceptive therapy or pregnancy) at the time of their prior VTE, than in those with abnormal thrombophilia testing and/or an unprovoked event [138]; in the two subsequent retrospective studies, the presence or absence of a definable thrombophilia did not appear to influence the risk of recurrent pregnancy-associated VTE [140, 141]. Studies in nonpregnant patients have also demonstrated that thrombophilic abnormalities do not play an important role in determining the risk of recurrent VTE, despite being clear risk factors for a first episode of DVT or PE [142]. There was a suggestion in the two retrospective studies that women with a first VTE provoked by oral contraceptives or related to pregnancy might be at higher risk of recurrence in a subsequent pregnancy than those with an unprovoked event or VTE related to a transient non-hormonal risk factor [140, 141]. The latter findings are consistent with those from an observational administrative dataset from California [143].

The above data suggest that pregnant women with a single prior episode of VTE associated with a transient risk factor not related to pregnancy or use of estrogen are likely at lower risk of recurrent antepartum VTE compared to pregnant women with a history of unprovoked, pregnancy or estrogen-related VTE. The ACCP guidelines estimated the risk of recurrent antepartum VTE without prophylaxis to be 2 % in the first group and 8 % in the second group [23]. Current guidelines favor a strategy of antepartum clinical vigilance for those with a single prior episode of VTE associated with a transient risk factor not related to pregnancy or hormone use and antepartum LMWH with a history of unprovoked, pregnancy or estrogen-related VTE [17, 19, 20, 23]. However, as the available data have significant limitations, antepartum clinical vigilance may also acceptable for higher risk patients accepting of the risks of recurrence and for whom the burden of LMWH prophylaxis outweighs potential benefits. Similarly, women with a prior VTE associated with a transient risk factor not related to pregnancy or use of estrogen may benefit from antepartum prophylaxis if they have additional major risk factors for thrombosis. Although supportive data from clinical trials are lacking, postpartum prophylaxis for 6 weeks with prophylactic or intermediate dose LMWH or vitamin K antagonists targeted at INR 2.0–3.0 is generally recommended for all pregnant women with prior VTE not receiving long-term anticoagulants [17, 19, 20, 23].

*Prevention of VTE in pregnant women with thrombophilia and no prior VTE*

Thrombophilias are laboratory abnormalities associated with an increased risk of thrombosis and can be either inherited or acquired. The majority of studies that have examined the risk of VTE in pregnancy have focused on inherited thrombophilic mutations. Although it has been reported that approximately 50 % of pregnancy-associated VTE are associated with inherited thrombophilia; these abnormalities are very common and collectively are present in at least 15 % of the population [144, 145].

As shown in Table 5, in a systematic review of nine case control studies (n = 2526) that evaluated the association between thrombophilia and pregnancy-associated VTE, the highest risks were associated with homozygosity for factor V Leiden or the prothrombin G20210A variant [146]. Pregnant women with the most common heritable thrombophilias (i.e. heterozygosity for factor V Leiden or the prothrombin G20210A variant) had lower risks. Deficiencies of antithrombin, protein C, and protein S were associated with moderate risk increases. Estimated absolute VTE risks, calculated using the provided odds ratios and a background incidence of VTE during pregnancy of approximately 1/1000 deliveries, suggest a low thrombosis risk (0.5–1.2 % of affected pregnancies) for most of the inherited thrombophilias, except perhaps for homozygous carriers of the factor V Leiden or the prothrombin mutations, where the risk estimate is approximately 4 % (Table 5). However, these findings are limited by the fact that most of the included women would not have had a family history of VTE. A positive family history of VTE increases the risk for VTE two- to four-fold, depending on the number of affected relatives [147, 148] and thrombophilic subjects without a personal or family history of DVT or PE have lower rates of VTE than patients with thrombophilia and a positive family history [149]. Family-based cohort studies not included in the above-mentioned systematic review suggest that the risks of developing a first VTE during pregnancy and the postpartum period are two to four times greater than estimated in thrombophilic women without a positive family history (Table 5) [150–161]. However, it should be noted that many of the events occurred during the postpartum period and these risk estimates are very imprecise, particularly for the less common thrombophilias.

**Table 5:** Risks of pregnancy-related VTE in asymptomatic thrombophilic women

Thrombophilia	Estimated relative risk OR (95 %CI)^a^	Estimated absolute risk of VTE % of pregnancies (antepartum and postpartum) (95 % CI)
Antithrombin, protein C or protein S deficiency		
Family studies		4.1 (1.6–8.3)^b^ 3.2 (0.5–16.2) (total)^c^ 8.3 (1.5–35.4) (no prophylaxis)^c^
Antithrombin deficiency		
Non-family studies	4.7 (1.3–17.0)	0.7 (0.2–2.4)^a^
Family studies		3.0 (0.08–15.8)^b^ 8.3 (1.4–35.4)(total)^c^ 14.3 (2.6–51.3)(no prophylaxis)^c^
Protein C deficiency		
Non-family studies	4.8 (2.2–10.6)	0.7 (0.3–1.5)^a^
Family studies		1.7 (0.4–8.9)^b^ 0 (0–25.9) (total)^c^ 0 (0–79.4) (no prophylaxis)^c^
Protein S deficiency		
Non-family studies	3.2 (1.5–6.9)	0.5 (0.2–1.0)^a^
Family studies		6.6 (2.2–14.7)^b^ 0 (0–32.4) (total)^c^ 0 (0–48.9) (no prophylaxis)^c^
Factor V Leiden, heterozygous		
Non-family studies	8.3 (5.4–12.7)	1.2 (0.8–1.8)^a^
Family studies		3.1 (2.1–4.6)^d^
Factor V Leiden, homozygous		
Non-family studies	34.4 (9.9–120.1)	4.8 (1.4–16.8)^a^
Family studies		14.0 (6.3–25.8)^e^
Prothrombin G20201A, heterozygous		
Non-family studies	6.8 (2.5–18.8)	1.0 (0.3–2.6)^a^
Family studies		2.6 (0.9–5.6)^f^
Prothrombin G20201A, homozygous		
Non family history of studies	26.4 (1.2–559.3)	3.7 (0.2–78.3)^a^

^a^Estimated absolute risks are derived by multiplying the pooled odds ratios with their corresponding 95 % CIs from Robertson et al. [146] with the overall baseline VTE incidence (i.e. antepartum and until 6 weeks postpartum combined) of 1.4 per 1000 [23]

^b^Data from Friederich et al. [160]

^c^Data from Mahmoodi et al. [151]

^d^Data from Middeldorp et al. [152], Simioni et al. [156], Middeldorp et al. [157], Simioni et al. [158], Couturaud et al. [159]

^e^Data from Middeldorp et al. [152], Martinelli et al. [153], Tormene et al. [153]

^f^Data from Bank et al. [160], and Coppens et al. [161]

Acquired thrombophilias have been less well studied but repeated antiphospholipid antibody positivity (lupus anticoagulants [non-specific inhibitors], anticardiolipin antibodies, or anti-β2glycoprotein-I antibodies) is associated with an increased risk of VTE [162]. The risk or pregnancy-related VTE in women with antiphospholipid antibodies and no previous history of venous thrombosis is uncertain [163, 164].

There is considerable disagreement between current guidelines about the indication for antepartum thrombosis prophylaxis in pregnant women with deficiencies of antithrombin, protein C, or protein S. The inconsistency in recommendations likely results from the use of different risk thresholds for suggesting prophylaxis, uncertainty in risk estimates in recent studies, as described above, and concerns about VTE risks presented in older studies that suggested that these are high risk thrombophilias [165–167]. However, this data is somewhat problematic as these papers have methodologic limitations, including acceptance of non-objectively diagnosed outcome events, failure to clearly specify criteria for the diagnosis of VTE, including recurrent VTE episodes in women who already had had a VTE, and the potential for referral and recall bias, that have the potential to lead to an overestimation of risk.

*Prevention of pregnancy-associated VTE in patients with clinical risk factors*

Most studies that have assessed clinical and pregnancy-related risk factors for VTE have utilized a case control or cross-sectional design (Table 6) [3–5, 7, 87, 168–170]; although a few recent publications have used large databases to provide population-level absolute and relative risks for VTE [171, 172]. In methodologically stronger studies, most established risk factors have only a modest effect on VTE risk, with few increasing the absolute risk about 1 %. How combinations of independent risk factors might affect overall VTE risk has not been extensively studied and in most cases, it is unclear whether combinations result in additive or multiplicative risks. Further research in this area is required.

**Table 6:** Clinical risk factors for VTE as determined from case–control or cross-sectional studies

Risk factor	Adjusted OR/HR	95 % CI
Antepartum risk		
Immobility (strict bedrest for ≥1 week in the antepartum period) with pre-pregnancy BMI ≥25 kg/m^2^	62.3	11.5–337.0
Immobility (strict bedrest for ≥1 week in the antepartum period) with pre-pregnancy BMI <25 kg/m^2^	7.7	3.2–19.0
Assisted reproductive techniques—first trimester	4.6	2.9–7.2
Spontaneous twins	2.6	1.1–6.2
Antepartum hemorrhage	2.3	1.8–2.8
Smoking (10–30 cigarettes/d prior to or during pregnancy)	2.1	1.3–3.4
Pre-pregnancy BMI ≥ 25 kg/m^2^—no immobilization	1.8	1.3–2.4
Weight gain <7.0 kg	1.7	1.1–2.6
Postpartum risk		
Immobility (strict bedrest for ≥1 week in the antepartum period) with pre-pregnancy BMI ≥25 kg/m^2^	40.1	8.0–201.5
Postpartum infection (clinical signs/symptoms with fever and elevated white blood cell count) following vaginal delivery	20.2	6.4–63.5
Postpartum hemorrhage ≥1000 mL with surgery (curettage, evacuation of hematoma, or re-operation after cesarean section)	12.0	3.9–36.9
Immobility (strict bedrest for ≥1 week in the antepartum period) with pre-pregnancy BMI <25 kg/m^2^	10.8	4.0–28.8
Postpartum infection (clinical signs/symptoms with fever and elevated white blood cell count) following cesarean section	6.2	2.4–16.2
Pre-eclampsia with fetal growth restriction	5.8	2.1–16.0
Postpartum hemorrhage >1000 mL with no surgery	4.1	2.3–7.3
Fetal growth restriction (gestational age + sex-adjusted birth weight <2.5th percentile)	3.8	1.4–10.2
Smoking (10–30 cigarettes/d prior to or during pregnancy)	3.4	2.0–4.4
Pre-eclampsia	3.1	1.8–5.3
Hyperemesis	2.5	2.0–3.2
Pre-pregnancy BMI <25 kg/m^2^—no immobilization	2.4	1.7–3.3
Smoking (5–9 cigarettes/d prior to or during pregnancy)	2.0	1.1–3.7
Pre-pregnancy >25 kg/m^2^—no immobilization	1.8	1.3–2.4
Risk period not specified		
Systemic lupus erythematosus	8.7	5.8–13.0
Blood transfusion	7.6	6.2–9.4
Heart disease	7.1	6.2–8.3
Sickle cell disease	6.7	4.4–10.1
Multiple pregnancy	4.2	1.8–9.7
BMI ≥30 kg/m^2^	5.3	2.1–13.5
Assisted reproductive techniques	1.8	1.4–2.3
Anemia	2.6	2.2–2.9
Diabetes	2.0	1.4–2.7
Hypertension	1.8	1.4–2.3
Weight gain >21 kg (vs. 7–21 kg)	1.6	1.1–2.6
Parity > 1	1.5	1.1–1.9

Data from: Lindqvist et al. [3], Simpson et al. [4], James et al. [5], Jacobsen et al. [7], Knight et al. [86], Henriksson et al. [168], Jacobsen et al. [169]

*BMI* body mass index

*Prevention of pregnancy-associated VTE following cesarean delivery*

Several observational studies have assessed the risk of VTE after cesarean delivery. Small prospective studies in which patients underwent screening ultrasounds following cesarean section and were then followed post-discharge for at least 6 weeks reported symptomatic VTE event rates of 0 (95 % CI 0–6.1 %) [173] and 0.5 % (95 % CI 0.1–2.8 %) [174]. The latter is consistent with estimates based on hospital discharge data that antedate the use of thrombosis prophylaxis [1, 175]. Emergency cesarean delivery approximately doubles the risk of VTE [7, 169, 172].

In the Cochrane systematic review mentioned above, four (840 women) of the nine included trials that examined prophylaxis following cesarean delivery compared heparin (UFH or LMWH) with placebo [119]. There was no evidence that using any form of heparin following delivery reduced the risk of maternal VTE (risk ratio [RR] vs no heparin for symptomatic events of 1.30; 95 % CI 0.39–4.27) and the authors concluded there was insufficient evidence on which to base recommendations.

### **Guidance Statement**

*Note: Given the uncertainty around optimal prophylactic strategies, all women should be provided with the opportunity to participate in shared decision making regarding this intervention, including a discussion of VTE risks, potential benefits (reduction in VTE risk) and drawbacks (risks of bleeding and localized skin reactions; cost; potential limitation of analgesic options at the time of delivery; anxiety associated with injections) of prophylaxis along with their values and preferences. Physicians and patients (and, perhaps, societies) with a lower threshold for recurrent VTE may choose a more aggressive anticoagulant strategy than recommended, whereas withholding prophylaxis may be appropriate in those who are willing to accept a higher risk of recurrence in order to forgo the drawbacks associated with prophylaxis.*

### General Comments

- *All pregnant women at risk of VTE should be educated about the signs and symptoms of DVT and PE and the need to seek urgent medical attention should they develop. Objective testing is mandatory if symptoms suspicious of DVT or PE occur.*
- *All women should undergo an individualized risk assessment for VTE prior to pregnancy, once pregnancy is achieved and throughout pregnancy as new clinical situations arise.*
- *When considering the use of thrombosis prophylaxis during pregnancy and/or the postpartum period, the absolute risk of VTE, the risk reduction with prophylaxis, drawbacks of prophylaxis, and the woman’s values and preferences should all be taken into account. Given the limitations of the available data, clinical vigilance rather than prophylaxis may also be acceptable for patients accepting the VTE risks quoted above and for whom the burden of LMWH prophylaxis outweighs potential benefits.*
- *If the decision is made to use antepartum prophylaxis, it should be initiated early in pregnancy.*
- *Six weeks of postpartum prophylaxis is recommended in patients with prior VTE and those with some thrombophilias. A shorter course of postpartum prophylaxis (until discharge or for 1–2 weeks post discharge) is suggested for women with transient risk factors.*

### *Prevention of recurrent VTE*

- *Pregnant women with prior VTE who are not receiving long-term anticoagulation should receive 6 weeks of postpartum prophylaxis.*
- *Antepartum prophylaxis should be considered in pregnant women with prior unprovoked VTE or pregnancy- or estrogen-related VTE not receiving long-term anticoagulation.*

### *Prevention of VTE in women with thrombophilia and no prior VTE*

- *Asymptomatic women who are homozygous for the factor V Leiden mutation or prothrombin gene mutation and who have a family history of VTE should receive antepartum and postpartum prophylaxis.*
- *Consideration should be given to providing postpartum prophylaxis in asymptomatic women who are heterozygous for the factor V Leiden mutation or prothrombin gene mutation or who have protein C or protein S deficiency and who have a family history of VTE.*
- *Given the variability in the VTE risk estimates for asymptomatic women with antithrombin deficiency and a family history of VTE, either a strategy of antepartum and postpartum prophylaxis or postpartum prophylaxis alone is reasonable.*
- *Asymptomatic women who are homozygous for the factor V Leiden mutation or prothrombin gene mutation and who have no family history of VTE should receive postpartum prophylaxis.*
- *Asymptomatic women with all other thrombophilias who do not have a family history of VTE do not require prophylaxis, in the absence of other risk factors. However, given the variability in the VTE risk estimates for asymptomatic women with antithrombin deficiency and no family history of VTE, consideration could also be given to utilizing postpartum prophylaxis in these patients.*

### *Prevention of VTE in women with clinical risk factors*

- *Antepartum prophylaxis should be provided to immobilized (strict bedrest) women with a pre-pregnancy BMI of at least 25 kg/m*^2^*and to those with a prior history of VTE regardless of their BMI. Consideration should be given to providing prophylaxis during antepartum immobilization (as defined above) to women with a lower body mass index who have other significant comorbidities (e.g. systemic lupus erythematosus, sickle cell disease, heart disease) associated with an increased risk of VTE or a thrombophilia.*
- *Consideration should be given to providing postpartum prophylaxis while in hospital to women with a history of antepartum immobilization (as defined above) for at least 7 days and to those who are immobilized postpartum who have a known thrombophilia or significant medical comorbidity*

### *Prevention of VTE in after cesarean delivery*

- *Prophylaxis should be provided after cesarean delivery to women with the following risk factors:*
  - *One or more of prior VTE, a history of antepartum immobilization (strict bedrest for at least 1 week), significant postpartum infection, postpartum hemorrhage of at least 1000 mL requiring re-operation, pre-eclampsia with growth restriction, significant medical co-morbidities (systemic lupus erythematosis, heart disease, or sickle cell disease) or a known thrombophilia.*
  - *Two or more of (or one or more in the setting of emergency cesarean delivery) of postpartum hemorrhage of at least 1000 mL that does not require re-operation, BMI >30 kg/m*^2^*, fetal growth restriction, pre-eclampsia, multiple pregnancy and tobacco use during pregnancy (at least 10 cigarettes per day)*

5. How is peripartum anticoagulation managed?

The delivery options in women using anticoagulants are best considered by a multidisciplinary team in order to minimize the risks of maternal hemorrhage, epidural hematoma, and VTE around the time of delivery. In a systematic review of 2777 pregnancies in which LMWH was utilized in either therapeutic or prophylactic doses, postpartum hemorrhage of greater than 500 mL occurred in 26 pregnancies (0.94 %; 95 % CI 0.61–1.37 %) and wound hematoma in 17 pregnancies (0.61 %, 95 % CI 0.36–0.98 %) [59]. A more recent systematic review of 18 studies that focused solely on pregnant women (n = 981) receiving treatment for acute VTE during pregnancy reported an incidence of major bleeding during the first 24 h after delivery of 1.90 % (95 % CI 0.80–3.60 %) [60]. Although epidural hematomas in obstetrical patients receiving epidural analgesia/anesthesia are rare, with an estimated incidence of less than 1 in 150,000 [176]; the potential complications are devastating and include permanent neurologic dysfunction.

Depending on local practice, delivery options include spontaneous labor and delivery, induction of labor, and planned cesarean delivery. Induction of labor may help to avoid an unwanted anticoagulant effect during delivery (especially with neuroaxial anesthesia) in women receiving LMWH. Current guideline recommendations for management of anticoagulant therapy around the time of delivery are outlined in Table 2D. Anesthesia and obstetrical guidelines agree that 24 h should pass between the last dose of therapeutic LMWH and insertion of a neuroaxial catheter [17, 19–23, 177]. For prophylactic LMWH, catheter insertion should occur no sooner than 10–12 h after the last LMWH dose [17, 19–23, 177].

At some centers, women are converted from therapeutic adjusted-dose LMWH to subcutaneous twice daily therapeutic dose UFH in the last month of pregnancy. However, therapeutic doses of subcutaneous UFH may cause a persistent anticoagulant effect. One study reported that six of 11 women receiving subcutaneous UFH during pregnancy had an elevated aPTT at delivery despite discontinuing their injections at the onset of labour to 12 h prior to elective induction [178].

Patients should be instructed to withhold their injections if they believe that they have entered labor spontaneously. In centers with laboratory support that allows for rapid assessment of heparin levels, testing can be considered to guide anesthetic and surgical management; otherwise time since last injection should be used. If anticoagulation precludes regional techniques, alternative analgesic options include intravenous analgesia or general anesthesia for cesarean delivery [20–22].

The potential increased risk of wound hematoma after cesarean delivery in patients receiving anticoagulant therapy has led to the suggestion that wound drains and closure techniques that allow easy hematoma drainage be considered in this population [179]. If bleeding occurs that is considered secondary to LMWH rather than an obstetric cause, protamine sulfate may provide partial neutralization [180].

Women diagnosed with proximal DVT or PE within two to 4 weeks of delivery are at very high risk for recurrent VTE with prolonged anticoagulant cessation [181, 182]. A strategy involving planned delivery with transition to intravenous UFH will minimize time off therapeutic anticoagulation [22, 23]. Discontinuation of intravenous UFH four to 6 h prior to the expected time of delivery or epidural insertion with a repeat activated partial thromboplastin time drawn after 4 h to confirm normalization will ensure that there is no residual anticoagulant effect. For the highest risk patients (e.g. VTE within 2 weeks), consideration can be given to insertion of a temporary inferior vena caval filter that can be removed postpartum.

Anticoagulants should be recommenced post-delivery as soon as adequate hemostasis is assured. Guidelines generally recommend resumption of prophylactic LMWH four to 12 h following delivery [17, 19]; and not sooner than 4 h after epidural catheter removal (with a longer delay for bloody or traumatic neuroaxial procedures) [177]. There are no definitive recommendations for resumption of full-dose LMWH following epidural catheter removal; however, it appears safe to do so 24 h of catheter removal (again, with a delay if placement was bloody or traumatic) [19, 22]. The timing of resumption of postpartum vitamin K antagonists for patients who choose this option remains controversial; some guidelines recommend a delay of at least 5 days [21, 22], although this recommendation appears based on the results of a single centre retrospective audit [183]. Once an INR of at least 2.0 is achieved, bridging LMWH can be discontinued.

### **Guidance Statement**

- *All pregnant women receiving anticoagulants should have an individualized delivery plan that addresses obstetrical, anesthetic and thrombotic concerns.*
- *All pregnant women should be advised to discontinue anticoagulant therapy upon the onset of spontaneous labor.*
- *If there is a planned delivery, therapeutic LMWH should be discontinued at least 24 h prior to the expected time of epidural analgesia or delivery. Prophylactic LMWH should be stopped at least 10–12 h prior to epidural analgesia.*
- *For planned deliveries, intravenously administered unfractionated heparin should be stopped at 4–6 h prior to the expected time of epidural analgesia or delivery and the aPTT checked to ensure normalization. For therapeutic doses of unfractionated heparin administered subcutaneously, the last dose should be given no sooner than 12 h and preferably closer to 24 h prior to expected time of epidural analgesia or delivery and the aPTT checked to ensure normalization. Guidelines differ in their requirement for a delay prior to epidural analgesia in patients receiving prophylactic dose unfractionated heparin up to 10,000 units daily; when possible prophylactic unfractionated heparin should be discontinued 8–10 h prior to planned procedures.*
- *Prophylactic LMWH may be started/restarted 6–12 h after delivery (no sooner than 4 h after epidural catheter removal), as long as hemostasis is assured and there has not been a bloody or traumatic epidural. For prophylactic unfractionated heparin, the recommended time interval from epidural catheter removal is one to 8 h.*
- *Therapeutic LMWH may be started/restarted 24 h after delivery (no sooner than 24 h after epidural catheter removal), as long as hemostasis is assured and there has not been a bloody or traumatic epidural. Attainment of therapeutic levels of intravenous unfractionated heparin should be delayed for the same amount of time.*

## Conclusion

Women are at increased risk of VTE during pregnancy and the postpartum period. Treatment and prevention of VTE in this patient population is complicated by the need to consider fetal, as well as maternal, wellbeing when making management decisions. Although our knowledge of risk factors for pregnancy-related VTE and the safe and effective use of anticoagulants used to prevent and treat VTE in this population continues to increase, there are still important gaps and high quality research in this area should be a priority. In the interim, all women should be provided with the opportunity to participate in shared decision making regarding their management. To make the best decisions, absolute risks and potential benefits of interventions, guideline recommendations, and patient values and preferences should all be taken into account. Table 7 summarizes these guidance statements.

**Table 7:** Summary of guidance statements

Question	Guidance statement
1. What are the risks of anticoagulant use during pregnancy?	Physicians should counsel women receiving long-term therapy with vitamin K antagonists and the oral direct-acting anticoagulants about the fetal risks of these medications before pregnancy occurs LMWH is the drug of choice for treatment and prevention of VTE in pregnancy, except in patients with HIT, a history of HIT, or significant renal dysfunction. UFH is preferred in patients with significant renal dysfunction For women taking vitamin K antagonists, two options are available to reduce the risk of warfarin embryopathy. The first is to advise women to perform frequent pregnancy tests and substitute LMWH for warfarin once pregnancy is achieved and before 6 weeks gestation. Alternatively, LMWH or UFH can be substituted for vitamin K antagonists before conception is attempted Although the latter approach minimizes the risks of early miscarriage associated with vitamin K antagonist therapy, it lengthens the duration of exposure to LMWH or UFH and, therefore, is costly and exposes the patient to a greater burden of treatment associated with the use of injectable heparin therapy. Since warfarin embryopathy is unlikely to result from warfarin exposure before 6 weeks, the first option is usually favored by guidelines. Although the management of women who are receiving long-term therapy with oral direct thrombin and factor Xa inhibitors and attempting to conceive remains controversial, it has been suggested that these women should be converted to a coumarin or LMWH before conception is attempted; failing that, the switch should be made as soon as pregnancy is confirmed In pregnant women with severe cutaneous allergies to UFH or LMWH or with HIT or a history of HIT, danaparoid or fondaparinux (if danaparoid not available) may be used. Appropriate dosage and management of these anticoagulants around the time of delivery should be discussed with a hematologist or thrombosis specialist
2. What are the risks of anticoagulation in breastfeeding women?	UFH, LMWH, warfarin and acenocoumarol are safe for the breast-fed infant when administered to the nursing mother The oral direct thrombin and factor Xa inhibitors should not be used while breastfeeding
3. How is venous thromboembolism during pregnancy treated?	Outpatient treatment of VTE can be considered in patients who are clinically stable and have good cardiorespiratory reserve, no major risk factors for bleeding and good social support with easy access to medical care. Hospitalization is indicated in patients who are hemodynamically unstable or do not have good social support and those who have extensive VTE, or maternal co-morbidities that limit their tolerance of recurrent VTE or increase their risk of major bleeding LMWH is the preferred anticoagulant for most pregnant women with acute VTE. UFH should be used instead of LMWH in patients with GFR less than 30 mL/min. Intravenous UFH should be considered in patients who may require thrombolysis, surgery or urgent delivery If LMWH is used for treatment of acute VTE in pregnancy, the same weight-adjusted dosing regimen as in the nonpregnant population should be utilized (Table 3). Routine monitoring of LMWH dosing with anti-Xa LMWH is likely not required Thrombolytic therapy should be reserved for pregnant women with PE associated with life-threatening cardiorespiratory compromise or limb-threatening DVT Insertion of a temporary inferior vena caval filter should be considered in pregnant women with acute VTE and a contraindication to anticoagulant therapy Anticoagulant therapy for treatment of VTE during pregnancy should be continued throughout pregnancy and for at least six weeks postpartum for a minimum duration of three months
4. How is pregnancy-associated VTE prevented?	General comments All pregnant women at risk of VTE should be educated about the signs and symptoms of DVT and PE and the need to seek urgent medical attention should they develop. Objective testing is mandatory if symptoms suspicious of DVT or PE occur All women should undergo an individualized risk assessment for VTE prior to pregnancy, once pregnancy is achieved and throughout pregnancy as new clinical situations arise When considering the use of thrombosis prophylaxis during pregnancy and/or the postpartum period, the absolute risk of VTE, the risk reduction with prophylaxis, drawbacks of prophylaxis, and the woman’s values and preferences should all be taken into account. Given the limitations of the available data, clinical vigilance rather than prophylaxis may also be acceptable for patients accepting the VTE risks quoted above and for whom the burden of LMWH prophylaxis outweighs potential benefits If the decision is made to use antepartum prophylaxis, it should be initiated early in pregnancy Six weeks of postpartum prophylaxis is recommended in patients with prior VTE and those with some thrombophilias. A shorter course of postpartum prophylaxis (until discharge or for one to 2 weeks post discharge) is suggested for women with transient risk factors Prevention of Recurrent VTE Pregnant women with prior VTE who are not receiving long-term anticoagulation should receive 6 weeks of postpartum prophylaxis Antepartum prophylaxis should be considered in pregnant women with prior unprovoked VTE or pregnancy- or estrogen-related VTE not receiving long-term anticoagulation Prevention of VTE in Women with Thrombophilia and no Prior VTE Asymptomatic women who are homozygous for the factor V Leiden mutation or prothrombin gene mutation and who have a family history of VTE should receive antepartum and postpartum prophylaxis Consideration should be given to providing postpartum prophylaxis in asymptomatic women who are heterozygous for the factor V Leiden mutation or prothrombin gene mutation or who have protein C or protein S deficiency and who have a family history of VTE Given the variability in the VTE risk estimates for asymptomatic women with antithrombin deficiency and a family history of VTE, either a strategy of antepartum and postpartum prophylaxis or postpartum prophylaxis alone is reasonable Asymptomatic women who are homozygous for the factor V Leiden mutation or prothrombin gene mutation and who have no family history of VTE should receive postpartum prophylaxis Asymptomatic women with all other thrombophilias who do not have a family history of VTE do not require prophylaxis, in the absence of other risk factors. However, given the variability in the VTE risk estimates for asymptomatic women with antithrombin deficiency and no family history of VTE, consideration could also be given to utilizing postpartum prophylaxis in these patients Prevention of VTE in women with clinical risk factors Antepartum prophylaxis should be provided to immobilized (strict bedrest) women with a pre-pregnancy BMI of at least 25 kg/m^2^ and to those with a prior history of VTE regardless of their BMI. Consideration should be given to providing prophylaxis during antepartum immobilization (as defined above) to women with a lower body mass index who have other significant comorbidities (e.g. systemic lupus erythematosus, sickle cell disease, heart disease) associated with an increased risk of VTE or a thrombophilia Consideration should be given to providing postpartum prophylaxis while in hospital to women with a history of antepartum immobilization (as defined above) for at least 7 days and to those who are immobilized postpartum who have a known thrombophilia or significant medical comorbidity Prevention of VTE in after Cesarean Delivery Prophylaxis should be provided after cesarean delivery to women with the following risk factors: One or more of prior VTE, a history of antepartum immobilization (strict bedrest for at least 1 week), significant postpartum infection, postpartum hemorrhage of at least 1000 mL requiring re-operation, pre-eclampsia with growth restriction, significant medical co-morbidities (systemic lupus erythematosis, heart disease, or sickle cell disease) or a known thrombophilia Two or more of (or one or more in the setting of emergency cesarean delivery) of postpartum hemorrhage of at least 1000 mL that does not require re-operation, BMI >30 kg/m^2^, fetal growth restriction, pre-eclampsia, multiple pregnancy and tobacco use during pregnancy (at least 10 cigarettes per day)
5. How is peripartum anticoagulation managed?	All pregnant women receiving anticoagulants should have an individualized delivery plan that addresses obstetrical, anesthetic and thrombotic concerns All pregnant women should be advised to discontinue anticoagulant therapy upon the onset of spontaneous labor If there is a planned delivery, therapeutic LMWH should be discontinued at least 24 h prior to the expected time of epidural analgesia or delivery. Prophylactic LMWH should be stopped at least 10–12 h prior to epidural analgesia For planned deliveries, intravenously administered unfractionated heparin should be stopped at 4–6 h prior to the expected time of epidural analgesia or delivery and the aPTT checked to ensure normalization. For therapeutic doses of unfractionated heparin administered subcutaneously, the last dose should be given no sooner than 12 h and preferably closer to 24 h prior to expected time of epidural analgesia or delivery and the aPTT checked to ensure normalization. Guidelines differ in their requirement for a delay prior to epidural analgesia in patients receiving prophylactic dose unfractionated heparin up to 10,000 units daily; when possible prophylactic unfractionated heparin should be discontinued 8–10 h prior to planned procedures Prophylactic LMWH may be started/restarted 6–12 h after delivery (no sooner than 4 h after epidural catheter removal), as long as hemostasis is assured and there has not been a bloody or traumatic epidural. For prophylactic unfractionated heparin, the recommended time interval from epidural catheter removal is one to 8 h Therapeutic LMWH may be started/restarted 24 h after delivery (no sooner than 24 h after epidural catheter removal), as long as hemostasis is assured and there has not been a bloody or traumatic epidural. Attainment of therapeutic levels of intravenous unfractionated heparin should be delayed for the same amount of time
